# Berry Phenolic and Volatile Extracts Inhibit Pro-Inflammatory Cytokine Secretion in LPS-Stimulated RAW264.7 Cells through Suppression of NF-κB Signaling Pathway

**DOI:** 10.3390/antiox9090871

**Published:** 2020-09-15

**Authors:** Inah Gu, Cindi Brownmiller, Nathan B. Stebbins, Andy Mauromoustakos, Luke Howard, Sun-Ok Lee

**Affiliations:** 1 Department of Food Science, University of Arkansas, 2650 N. Young Ave., Fayetteville, AR 72704, USA; inahgu@uark.edu (I.G.); cbrownm@uark.edu (C.B.); Nathan.Stebbins@tyson.com (N.B.S.); lukeh@uark.edu (L.H.); 2 Agricultural Statistics Laboratory, University of Arkansas, Fayetteville, AR 72701, USA; amauro@uark.edu

**Keywords:** berry volatiles, berry phenolics, anti-inflammatory effect, inflammatory cytokines, nuclear factor-kappa B

## Abstract

Berries are a rich source of phytochemicals, especially phenolics well known for protective activity against many chronic diseases. Berries also contain a complex mixture of volatile compounds that are responsible for the unique aromas of berries. However, there is very limited information on the composition and potential health benefits of berry volatiles. In this study, we isolated phenolic and volatile fractions from six common berries and characterized them by HPLC/HPLC-MS and GC/GC-MS, respectively. Berry phenolic and volatile fractions were evaluated for an anti-inflammatory effect using lipopolysaccharide (LPS)-stimulated RAW264.7 macrophage cells by measuring levels of pro-inflammatory cytokines and the nuclear factor-kappa B (NF-κB) signaling pathway. Results showed that LPS-induced excessive production of nitric oxide (NO), prostaglandin E_2_ (PGE_2_), cyclooxygenase-2 (COX-2), interleukin-6 (IL-6) and tumor necrosis factor-α (TNF-α), which were inhibited by berry phenolic and volatile extracts. Moreover, berry phenolic and volatile extracts reduced the nuclear translocation of NF-κB by blocking the phosphorylation of p65 and degradation of IκBα. These findings showed that berry volatiles from six berries had comparable anti-inflammatory effects to berry phenolics through the suppression of pro-inflammatory mediators and cytokines expression via NF-κB down-regulation, despite being present in the fruit at a lower concentration.

## 1. Introduction

Berries, among the most common fruits in the human diet (strawberry, blueberry, raspberry, blackberry and cranberry in the United States), are rich in natural compounds, such as minerals, vitamins, dietary fibers, but also contain phytochemicals such as polyphenols and volatiles [1,2]. Phenolic compounds, consisting of benzene rings with single or multiple hydroxyl substituents, are produced as secondary metabolites in plants [3,4]. There have been many studies that found significant beneficial health effects of berry phenolics including antioxidant, anti-inflammatory, anticancer, anti-obesity and cardiovascular disease-preventing effects in many studies [5,6,7,8,9,10].

A class of phytochemicals found in berries that have received little attention for their health-promoting properties are volatile compounds. Volatiles, compounds with high vapor pressure resulting from a low boiling point are present in plants at low concentrations (ppb to ppm). They are responsible for the unique aroma and flavor of berries, playing an important role in sensory perception [11,12]. Fruit volatile compounds mainly comprise diverse classes of chemicals, including acids, esters, alcohols, aldehydes, ketones, furans, lactones and terpenes [13]. Aroma is a complex mixture of many volatile compounds. As berry consumption of various products including fruits, juice, puree, jams and wine has increased due to its nutritional value and many health beneficial effects, many studies have identified aroma/flavor volatile compounds in products that impact consumer acceptability and consumption [14,15]. However, there is still very limited information of the chemical composition and health beneficial effects of berry volatiles. Volatiles play an important role in defense against abiotic and biotic stresses [16]. Plant essential oils, composed primarily of terpenes, are known to play an important role in health-promotion through anticancer, antioxidant, antiviral, antidiabetic and anti-inflammatory properties [17].

Inflammation is a defensive response in our body to fight infections such as chemical stimuli and toxins [10,18]. However, if chronic inflammatory conditions are extended, it can lead to many severe chronic diseases, including diabetes, cardiovascular diseases and cancer [19,20,21,22]. Overproduction of pro-inflammatory factors including nitric oxide (NO), prostaglandin E_2_ (PGE_2_) and cyclooxygenase-2 (COX-2) and cytokines such as tumor necrosis factor-α (TNF-α) and interleukin-6 (IL-6) increase the inflammatory response [23,24]. Furthermore, they activate a transcription factor such as nuclear factor-kappa B (NF-κB), which accelerates pathogenic inflammation and the development of chronic diseases. 

In this study, the objectives were to characterize phenolic and volatile fractions from six berries for chemical composition and investigate their anti-inflammatory effect using a lipopolysaccharide (LPS)-stimulated RAW264.7 macrophage cell model by measuring expression levels of key pro-inflammatory cytokines and the nuclear factor-kappa B (NF-κB).

## 2. Materials and Methods

### 2.1. Reagents

Lipopolysaccharides (LPS) from Escherichia coli O111:B4 were purchased from Sigma-Aldrich (St. Louis, MO, USA). Dulbecco’s modified Eagle’s medium (DMEM), fetal bovine serum (FBS), phosphate buffered saline (PBS, pH 7.2) and other cell culture reagents were purchased from Thermo Fisher Scientific (Waltham, MA, USA). 3-(4,5-dimethylthiazol-2-yl)-5-(3 carboxymethoxyphenyl)-2-(4-sulfophenyl)-2H-tetrazolium (MTS) and the Griess reagent system were obtained from Promega Co. (Madison, WI, USA). Antibodies against phosphorylation specific NF-κB p65, IκBα, COX-2, β-actin and cyclophilin B (CyPB) were purchased from Cell Signaling Technology, Inc. (Danvers, MA, USA).

### 2.2. Plant Materials

Fresh blackberries, blueberries, red raspberries and strawberries were obtained from a local supermarket (Sam’s Club, Fayetteville, AR, USA). Frozen cranberries were purchased from Northwest Wild Foods (Burlington, WA, USA), and frozen black raspberries (Stahlbush Island Farms, Corvallis, OR, USA) were purchased from a local supermarket (Ozark Natural Foods, Fayetteville, AR, USA).

### 2.3. Extraction of Berry Phenolics

For berry phenolic extracts, 50 g berries was homogenized in 50 mL methanol/water/formic acid (60:37:3 *v*/*v*/*v*) solution for 1 min using a Euro Turrax T18 Tissuemizer (Tekmar-Dohrman Corp, Mason, OH, USA). Homogenates were centrifuged for 5 min at 10,864× *g* (Beckman-Coulter Allegra X-22R, Beckman Coulter, Brea, CA, USA), and the pellet was re-extracted with 50 mL acetone/water/acetic acid (70:29.5:0.5 *v*/*v*/*v*) solution. This extraction was repeated twice with homogenates. After centrifuging homogenates again for 5 min at 10,864× *g*, collected supernatants were dried at 40 °C using a Buchi Rotary Evaporator R-114 (Buchi, Flawil, Switzerland). The dried sample was resolubilized in 50 mL deionized water for further purifying with solid phase extraction using Sep-Pak C18 20 cc Vac Cartridge (5 g sorbent per cartridge, 55–105 μm particle size, Waters Corp, Milford, MA, USA). After activating cartridges by eluting 40 mL methanol, followed by 80 mL water, the reconstituted sample was loaded onto the column cartridge. The sample was eluted with 80 mL 70% ethanol after rinsing with 60 mL water. The eluent was evaporated to dryness at 40 °C and reconstituted with 50 mL deionized water.

### 2.4. HPLC Analysis of Berry Phenolics

The purified phenolic fractions were evaluated by high-performance liquid chromatography (HPLC) using a Waters HPLC system equipped with a model 600 pump, a model 717 Plus autosampler and a model 996 photodiode array detector. Samples (50 μL) were analyzed, and the anthocyanins and hydroxycinnamic acids were identified and quantified as described by Cho et al. [25]. Compounds were separated on a 4.6 × 250 mm Symmetry C18 column (Waters Corp, Milford, MA, USA) preceded by a 3.9 × 20 mm Symmetry C18 guard column. The mobile phase consisted of a linear gradient of 5% formic acid (A) and methanol (B), which ran from 2% B to 60% B for 60 min at a flow rate of 1 mL/min. Ellagitannins and flavonols were separated on a 4.6 × 250 mm Aqua C18 column (Phenomenex, Torrance, CA, USA) preceded by a 3.0 × 4.0 mm octadecylsilyl (ODS) C18 guard column (Phenomenex) as described in Hager et al. [26] and Cho et al. [27]. The mobile phase consisted of a gradient of 2% acetic acid (A) and 0.5% acetic acid in water and acetonitrile (50:50 v/v, B), which ran from 10% B to 55% B in 50 min and from 55% B to 100% B in 10 min at a flow rate of 1 mL per min. Each system was equilibrated for 20 min at the initial gradient prior to each injection. Detection wavelengths of 280, 320, 360 and 510 nm were used for ellagitannins, hydroxycinnamic acids, flavonols and anthocyanins, respectively. Ellagitannins were quantified as mg ellagic acid equivalents per 100 g fresh weight (FW), hydroxycinnamic acids were quantified as mg chlorogenic acid equivalents per 100 g FW and flavonols were quantified as mg rutin equivalents per 100 g FW. Individual anthocyanin monoglucosides and acylated anthocyanin derivatives were quantified as delphinidin, cyanidin, petunidin, peonidin and malvidin glucoside equivalents, with results expressed as mg anthocyanin equivalents per 100 g FW.

### 2.5. Extraction of Berry Volatiles

A low temperature hydrodistillation technique under reduced pressure was used to extract volatiles. This technique was used in order to avoid hydrolysis, polymerization and decomposition of volatiles, which can occur at higher temperatures. For berry volatile extracts, 300 g berries, 300 mL deionized water and 100 g NaCl were blended using a Waring blender for 1 min. The homogenate was vacuum distilled in a 50 °C water bath and a 0 °C condenser for 30 min using a rotary evaporator. Berry volatile extract (200 mL) was collected in an ice bath incubated flask and stored at −20 °C until use. Solid phase micro-extraction (SPME) of volatiles was performed using a 85 μm, CAR/PDMS, Stableflex, 24 Ga, Manual Supelco (Bellefonte, PA, USA) SPME fiber as previously described [28].

### 2.6. GC/GC-MS Analysis of Berry Volatiles

Gas chromatography quantification of volatiles desorbed from SPME fibers was performed as previously described [28].

### 2.7. Determination of Total Phenolics, Total Anthocyanins and Antioxidant Capacity

The total phenolic contents of berry phenolic and volatile extracts were determined by the Folin—Ciocalteu assay [29] using a gallic acid standard curve. Absorbance was measured at 760 nm using a Hewlett Packard (Palo Alto, CA, USA) 8452A diode-array spectrophotometer, with results expressed as mg gallic acid equivalents per 100 g FW. Total anthocyanins were determined using the method of Giusti and Wrolstad [30]. Absorbance was measured using the same spectrophotometer described above at 520 and 700 nm in buffers at pH 1.0 and 4.5 using A = (A520 − A700)pH 1.0 − (A520 − A700)pH 4.5. Cyanidin 3-glucoside (C3G) with a molecular weight of 449.2 g/mol and a molar extinction coefficient of 26,900 L × cm^−1^ × mol^−1^ was used as standard, with results expressed as mg C3G equivalents per 100g FW. The free radical scavenging activity of phenolic and volatile extracts was measured using the 2,2-diphenyl-1-picrylhydrazyl (DPPH) assay adapted by Akkari et al. [31]. A volume of 1.4 mL solution of DPPH in methanol (0.1 mM) was added to 0.1 mL of each phenolic or volatile extract at different concentrations (10, 20, 40, 80 and 160× dilutions of the purified extracts), incubated in a dark room for 30 min, then the absorbance was measured at 517 nm. Radical-scavenging activity was expressed as μM of trolox equivalents (TE) per 100 g FW for phenolic extracts and μM per kg FW for volatile extracts.

### 2.8. Cell Culture and Treatment

RAW264.7 (ATCC^®^ TIB-71™) macrophage cells from mice were purchased from American type culture collection (ATCC, Manassas, VA, USA). RAW264.7 were cultured in DMEM supplemented with 10% FBS, 2 mM L-glutamine, 100U/mL penicillin and 100µg/mL streptomycin. The cells were maintained in a humidified 5% CO2 incubator (VWR^®^ Water Jacketed CO2 incubator, VWR International, PA, USA) at 37 ℃. For experiments, RAW264.7 cells were seeded in 96-well plates at a density of 1.0 × 104 cells/well overnight (16 h) and pretreated with 3 different dilutions (50, 100 and 200-fold) of 6 berry (cranberry, black raspberry, red raspberry, strawberry, blueberry and blackberry) polyphenolic and volatile extracts for 1 h. Berry phenolic and volatile extracts were dissolved in Tween 80 (0.03% final concentration) with DMEM before treatment. The cytotoxicity of Tween 80 itself on RAW264.7 cells were preliminarily tested, and Tween 80 at the concentration 0.05% or below showed no cytotoxic effect (data not shown). Cells were then incubated with or without LPS (100 ng/mL) for 24 h.

### 2.9. Cell Viability Assay and Nitric Oxide Determination

The effect of berry phenolic and volatile extracts on cell viability was measured using the CellTiter 96 AQueousOne Solution Cell Proliferation Assay (MTS assay) (Promega Co., Madison, WI, USA) according to the manufacturer’s instruction. RAW264.7 cells were plated at a density of 1.0 × 104 cells/well in 96-well plates containing 100 µL of culture medium, and incubated in a 37 °C, 5% CO_2_ incubator overnight (16 h). After overnight incubation, the cells were pretreated with 3 different dilutions of berry phenolic and volatile extracts (50, 100 and 200-fold) for 1 h. The cells were then stimulated by 100 ng/mL of LPS for 24 h. After 24 h, an aliquot of 20 µL One Solution Reagent (MTS) was added into each well, and the cells were further incubated for an additional 2 h. The absorbance was measured at 490 nm with a microplate reader (Synergy HT Multi-Mode Microplate Reader, BioTek Instruments, Inc. Winooski, VT, USA). The quantity of the formazan product, measured as the absorbance at 490 nm, indicates the proportion of the number of living cells in each well. Cell viability was calculated as the percentage of surviving cells over control cells.

Nitric oxide level of RAW264.7 cells treated with berry phenolic and volatile extracts with or without LPS were evaluated using a Griess reaction [32]. After LPS (100 ng/mL) stimulation for 24 h, the plate was centrifuged for 1 min at 140× *g*. Culture supernatants (50 µL) were collected to measure nitrite content. An equal volume (50 µL) of Griess reagents (Promega Co., Madison, WI, USA) was added and incubated at room temperature for each 10 min (0.1% N-1-napthylethylendiamine dihydrochloride in water and 1% sulfanilamide in 5% phosphoric acid). The absorbance was measured at 540 nm using a microplate reader. A culture medium was used as a blank in the experiment. Nitrite level produced as an indicator of NO synthesis was calculated against a sodium nitrite standard curve.

### 2.10. ELISA for IL-6, TNF-α and PGE_2_

LPS-stimulated IL-6, TNF-α and PGE_2_ production on RAW264.7, which were pretreated with berry phenolic and volatile extracts, was measured by the RayBio^®^ Mouse IL-6 and TNF-α Enzyme-linked immunosorbent assay (ELISA) Kits (RayBiotech Inc., Norcross, GA, USA) and PGE_2_ ELISA Kit (Cayman Chemical, Ann Arbor, MI, USA) according to the manufacturer’s instructions.

### 2.11. Western Blot Analysis

Cells were seeded in 90 mm dishes at a density of 2.0 × 106 cells/dish, incubated overnight (16 h) and pretreated with six 50-fold diluted berry phenolic or volatile extracts for 1 h. After 24 h LPS (100 ng/mL) stimulation, cells were rinsed with PBS twice and lysed with RIPA lysis and extraction buffer (Thermo Fisher Scientific, Waltham, MA, USA) with a protease inhibitor at 4 °C for 1 h. The lysate was centrifuged at 9240× *g* for 15 min at 4 °C, and supernatants were collected. The concentration of protein in each lysate of samples was measured by using BCA (bicinchoninic acid) protein assay kits (Thermo Fisher Scientific, Waltham, MA, USA). A total of 40 μg protein per sample was loaded and separated by using 10% sodium dodecyl sulfate-polyacrylamide gel electrophoresis (SDS-PAGE). The separated proteins were transferred to polyvinylidene difluoride (PVDF). The membrane was blocked with 5% bovine serum albumin (BSA) in Tris-buffered saline containing 0.1% Tween 20 (TBST) for 1 h. After incubating with primary antibodies overnight at 4 °C, the membrane was washed with TBST for 10 min three times and incubated with horseradish peroxidase-linked antibody (Cell Signaling Technology, Inc., Danvers, MA, USA), for 1 h. The membrane was washed again with TBST three times, and the signals were developed using an enhanced chemiluminescent (ECL) western blotting detection kit (Bio-Rad Laboratories, Inc., Hercules, CA, USA). The density of protein on bands was quantified with ImageJ software (U.S. National Institutes of Health, Bethesda, MD, USA). The signals were captured on x-ray film (CL-XPosure™ Film, Thermo Fisher Scientific) which was developed in a dark room. The density of protein on bands were quantified with ImageJ software (U.S. National Institutes of Health, Bethesda, MD, USA).

### 2.12. Statistical Analysis

One-way analysis of variance (ANOVA) using JMP PRO Version 14.3 and SAS 9.4 (SAS Institute Inc., Cary, NC, USA) was used to determine differences in all experiments. Differences between means were determined using the Student’s *t*-test (α = 0.05). Data were presented as the mean ± standard error of the mean (SEM). All experiments were conducted in at least triplicate. Tukey’s multiple comparison was used to determine significant differences among treatments (*p* < 0.05).

## 3. Results

### 3.1. Composition of Berry Phenolic Extracts

The total phenolic and total anthocyanin contents of the six berry extracts are shown in Table 1. The phenolic composition of the six berry extracts determined by HPLC are presented in Tables S1–S6.

#### 3.1.1. Cranberries

The cranberry extract had a total phenolic content of 421.3 mg/100 g FW, which was much higher than the range of 120–221 mg/100 g FW previously reported for cranberry varieties [33,34]. The total anthocyanin content of 98.6 mg/100 g FW fell within the range of previously reported values 21–118 mg/100 g FW for cranberry varieties [33,34]. HPLC results found the extract to contain 96.5 mg/100 g FW anthocyanins, 18.4 mg/100 g FW phenolic acids, 10.1 mg/100 g FW flavonols and 1.4 mg/100 g FW procyanidins (Table S1). The individual phenolics in the extract were similar to previous reports [35,36,37], with the exception of procyanidins, whose concentration is underestimated by reverse phase HPLC analysis, due to the inability to separate procyanidins with a degree of polymerization greater than three.

#### 3.1.2. Black Raspberries

The black raspberry extract had a total phenolic content of 1186.4 mg/100 g FW, which was higher than the range of 890–1079 mg/100 g FW previously reported for black raspberry varieties [38]. The total anthocyanin content of 342.0 mg/100 g FW was lower than previously reported values 464–627 mg/100 g FW for black raspberry varieties [38]. HPLC results found the extract to contain 484.0 mg/100 g FW anthocyanins, 42.4 mg/100 g FW ellagitannins and 34.7 mg/100 g FW flavonols (Table S2). The individual phenolics in the extract were similar to previous reports [35,39].

#### 3.1.3. Red Raspberries

The red raspberry extract had a total phenolic content of 208.1 mg/100 g FW, which falls at the low end of the range of 192–512 mg/100 g FW reported for red raspberry varieties [40,41]. The total anthocyanin content of 19.1 mg/100 g FW was at the low end of the range of previously reported values 19–51 mg/100 g FW for red raspberry varieties [40]. HPLC results found the extract to contain 32.0 mg/100 g FW anthocyanins, 14.6 mg/100 g FW ellagitannins, 8.3 mg/100 g FW procyanidins and 3.1 mg/100 g FW flavonols (Table S3). The individual phenolics in the extract were similar to previous reports [35,42,43].

#### 3.1.4. Strawberries

The strawberry extract had a total phenolic content of 125.4 mg/100 g FW, which falls within the range of 43–273 mg/100 g FW reported for strawberry varieties [44,45]. The total anthocyanin content of 8.7 mg/100 g FW (Table 1) was lower than previously reported values 19–84 mg/100 g FW for strawberry varieties [44,45]. HPLC results found the extract to contain 22.6 mg/100 g FW anthocyanins, 5.1 mg/100g FW flavonols and 3.5 mg/100 g FW ellagitannins (Table S4). The individual phenolics in the extract were similar to previous reports [35,46,47].

#### 3.1.5. Blueberries

The blueberry extract had a total phenolic content of 389.5 mg/100 g FW, comparable to the range of values 227–583 mg/100 g FW previously reported for Southern highbush blueberry varieties [48,49]. The total anthocyanin content of 184.9 mg/100 g FW was higher than previously reported values for Southern highbush blueberry varieties—35–157 mg/100 g FW [48,49]. According to the HPLC results, the extract contained 190.4 mg/100 g FW anthocyanins, 21.2 mg/100 g FW chlorogenic acid, 20.9 mg/100 g FW flavonols and 3.6 mg/100 g FW procyanidins (Table S5). The individual phenolics in the extract are similar to previous reports [25,35,50,51], with the exception of procyanidins, whose concentration is underestimated by reverse phase HPLC analysis, due to inability to separate procyanidins with a degree of polymerization greater than three.

#### 3.1.6. Blackberries

The blackberry extract had a total phenolic content of 386.2 mg/100g FW, which falls within the range of 275–650 mg/100 g FW reported for blackberry varieties [49]. The total anthocyanin content of 70.2 mg/100 g FW was lower than previously reported values 80–230 mg/100 g FW for blackberry varieties [49]. HPLC results found the extract to contain 99.1 mg/100 g FW anthocyanins, 16.0 mg/100 g FW ellagitannins, 15.2 mg/100 g FW flavonols and 7.0 mg/100 g FW procyanidins (Table S6). The individual phenolics in the extract are similar to previous reports [25,27,35,50,52].

### 3.2. Composition of Berry Volatile Extracts

The total volatiles in each berry extract are presented in Table 1, and the content of volatile classes present in each berry extract are presented in Table 2. The individual volatiles present in each berry extract are presented in Table S7.

#### 3.2.1. Cranberries

Thirty-five volatiles were identified in the cranberry extract, including 16 monoterpenes, 8 alcohols, 6 aldehydes, 2 esters, 2 ketones and 1 acid. The extract had a total volatile concentration of 2864.9 μg/kg. Monoterpenes were the predominant volatiles accounting for 60% of total volatiles, followed by esters (14%), acids (12%), alcohols (6%), aldehydes (6%) and ketones (2%). Major individual volatiles in the extract included α-terpineol (24%), eucalyptol (12%), 2-methylbutyric acid (12%), ethyl benzoate (10%), citronellol (9%) and linalool (7%). All of the major volatiles identified were previously reported in cranberries [53,54,55,56,57].

#### 3.2.2. Black Raspberries

Seventy-eight volatiles were identified in the black raspberry extract, including 28 monoterpenes, 10 alcohols, 9 aldehydes, 9 C13 norisoprenoids, 7 esters, 4 sesquiterpenes, 3 phenolics, 2 ketones, 2 lactones, 1 alkylbenzene, 1 furan, 1 hydrocarbon and 1 acid. The extract had a total volatile concentration of 8556.1 μg/kg. Monoterpenes were the predominant volatiles accounting for 61% of total volatiles, followed by alcohols (14%), esters (10%), aldehydes (8%) and acids (4%), while C13 norisoprenoids, ketones, sesquiterpenes, phenolics, lactones and ethers each accounted for < 2% of total volatiles. Major individual volatiles in the extract included (−)-myrtenol (23%), linalool (13%), α-terpineol (9%), 2-ethylhexanol (7%), cuminaldehyde (6%), and hexanoic acid, ethyl acetate and (+) myrtenol (each 4%). Volatiles in black raspberries have previously not been analyzed, but several of the major compounds—ethyl acetate, linalool, α-terpineol and (−)-myrtenol—were identified in black raspberry wine [58].

#### 3.2.3. Red Raspberries

Seventy-three volatiles were identified in the red raspberry extract, including 27 monoterpenes, 11 alcohols, 7 C13 norisoprenoids, 5 acids, 5 esters, 5 aldehydes, 4 lactones, 3 ketones, 2 phenolics, 2 furanones, 1 alkylbenzene and 1 hydrocarbon. The extract had a total volatile concentration of 2055.5 μg/kg. Monoterpenes were the predominant volatiles accounting for 47% of total volatiles, followed by acids (24%), C13 norisoprenoids (9%), aldehydes (5%), alcohols and phenolics (4% each), ketones (3%), esters (2%), while lactones, furans and hydrocarbons each accounted for < 2% of total volatiles. Major individual volatiles in the extract included myrtenol (21%), butanoic acid (15%), linalool and eugenol (each 7%), 3-methylbutanoic acid (5%), and α-ionone and vanillin (each 3%). All of the major volatiles identified were previously reported in red raspberries [59,60,61,62,63].

#### 3.2.4. Strawberries

Fifty-four volatiles were identified in the strawberry extract, including 13 monoterpenes, 12 esters, 6 alcohols, 6 ketones, 5 aldehydes, 3 acids, 2 phenolics, 2 C13 norisoprenoids, 1 lactone, 1 benzothiazole, 1 alkylbenzene, 1 hydrocarbon and 1 furan. The extract had a total volatile concentration of 2989.7 μg/kg. Monoterpenes were the predominant volatiles accounting for 43% of total volatiles, followed by acids (20%), esters (14%), furans (7%), aldehydes (5%), alcohols, ketones and alkylbenzene (3% each), while all other volatiles each accounted for < 1% of total volatiles. Major individual volatiles in the extract included myrtenol (35%), butanoic acid (7%), mesifuran (7%), ethyl butanoate (7%) and hexyl butanoate (4%). All of the major volatiles identified were previously reported in strawberries [64,65,66,67].

#### 3.2.5. Blueberries

Forty-six volatiles were identified in the blueberry extract, including 16 monoterpenes, 8 aldehydes, 7 alcohols, 5 esters, 2 acids, 2 ketones, 1 furan, 1 furanone, 2 C13 norisoprenoids, 1 phenolic and 1 sesquiterpene. The extract had a total volatile concentration of 1424.1 μg/kg. Monoterpenes were the predominant volatiles accounting for 45% of total volatiles, followed by alcohols (17%), aldehydes (8%), C13 norisoprenoids and esters (7% each), furans (5%) and ketones (4%), while acids, phenolics and sesquiterpenes each accounted for < 3% of total volatiles. Major volatiles in the extract included linalool (13%), linalool oxide (13%), phenylethyl alcohol (8%), 2-ethylhexanol (6%) and α-terpineol and beta ionone (each 4%). All of the major volatiles identified have previously been reported in blueberries [68,69,70,71].

#### 3.2.6. Blackberries

Sixty-one volatiles were identified in the blackberry extract, including 24 monoterpenes, 12 alcohols, 6 esters, 4 aldehydes, 4 ketones, 4 C13 norisoprenoids, 3 furans, 2 acids, 1 lactone and 1 phenolic. The extract had a total volatile concentration of 12,212.9 μg/kg, the highest volatile concentration among the six berries analyzed. Acids were the predominant volatiles accounting for 57% of total volatiles, followed by alcohols (18%), esters (10%), monoterpenes (10%), furans (2%), while ketones, C13 norisoprenoids, aldehydes, lactones and phenolics each accounted for < 2% of the total volatiles. Major individual volatiles in the extract included butanoic acid (39%), hexanoic acid (18%), 4-methyl-1-pentanol (10%), myrtenol (3%), 2- ethylhexanol (2%), isophorone (1%), limonene (1%) and 4-terpineol (1%). All of the major volatiles identified were previously reported in blackberries [59,72,73,74,75,76], with the exceptions of 2-ethylhexanol and isophorone.

### 3.3. Antioxidant Capacity of Phenolic and Volatile Extracts

DPPH radical scavenging activities of the six berry phenolic and volatile extracts are shown in Table 1. Due to abundant levels of phenolics, all berries analyzed possessed high antioxidant capacity measured using the DPPH assay. The antioxidant capacity of the berry phenolic extracts followed the order of black raspberry > blackberry > cranberry > blueberry > red raspberry > strawberry. Antioxidant capacity of the extracts was highly correlated with levels of total phenolics (rxy = 0.99) and total anthocyanins (rxy = 0.97). Due to anthocyanins being the most abundant class of polyphenols in the berries, levels of total anthocyanins were also highly correlated with levels of total phenolics (rxy = 0.98). On the other hand, the antioxidant activity of berry volatile extracts was nondetectible or very low.

### 3.4. Effect of Berry Phenolic and Volatile Extracts on RAW264.7 Cell Viability

**Figure 1:**
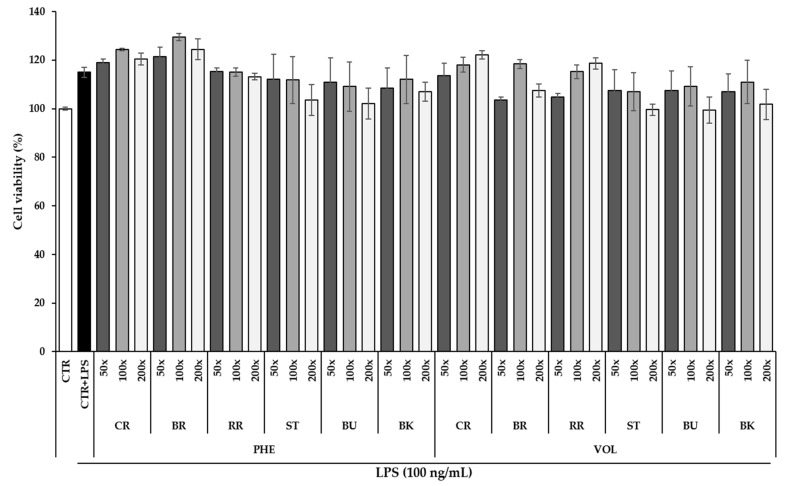
The cell viability of RAW264.7 cells treated with LPS and 6 berry phenolic and volatile extracts (50, 100 and 200-fold dilutions). Data are expressed as the mean ± SEM (*n* = 5). CTR = control; CR = cranberry; BR = black raspberry; RR = red raspberry; ST = strawberry; BU=blueberry; BK=blackberry. PHE = phenolics and VOL = volatiles.

Before investigating the anti-inflammatory effect, the cell cytotoxicity of berry phenolic and volatile extracts on RAW264.7 was evaluated by using MTS assay (Figure 1). There was no significant difference in cell viability between the control stimulated with LPS and the group pretreated with 3 different dilutions (50, 100 and 200-fold) of six different berry phenolic and volatile extracts. All berry phenolic and volatile extracts at tested dilutions showed no significant cytotoxicity on RAW264.7 macrophage cells. These results suggest that the anti-inflammatory effect of berry phenolic and volatile extracts found in RAW264.7 cells was not due to the cytotoxic effect. 

### 3.5. Effect of Berry Phenolic and Volatile Extracts on LPS-Induced NO Production

**Figure 2:**
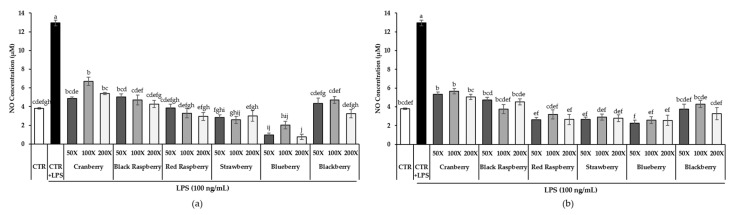
The production of nitric oxide (NO) in RAW264.7 cells treated with 6 (**a**) berry phenolic and (**b**) berry volatile extracts (50, 100 and 200-fold dilutions). Data are expressed as the mean ± SEM (*n* = 10). Means followed by a common letter are not significantly different by the Tukey’s test at the 5% level of significance. CTR = control.

To evaluate the inhibitory effect of berry phenolic and volatile extracts on LPS-stimulated nitric oxide production, NO concentration in the cultured medium was measured by using Griess reagent (Figure 2). The control with LPS-stimulation significantly increased the NO level compared to the control without LPS treatment (*p* < 0.05). Both berry phenolic and volatile extracts of three different dilutions of six berries inhibited LPS-induced NO production significantly by 48%–94% and 56%–82%, respectively, compared to the control stimulated with LPS (*p* < 0.05). There was no significant difference between the effect of berry phenolic and volatile extracts within each berry except blueberry. It showed that berry volatile extracts had a comparable inhibitory effect on LPS-induced NO production as berry phenolic extracts. In addition, there were no significant differences among the three different dilutions (50, 100 and 200-fold) within each berry phenolic or volatile extract in the NO production of RAW264.7 cells. Therefore, only a 50-fold dilution of berry phenolic and volatile extracts was used for further experiments. 

### 3.6. Effect of Berry Phenolic and Volatile Extracts on LPS-Stimulated PGE_2_/COX-2 Production

**Figure 3:**
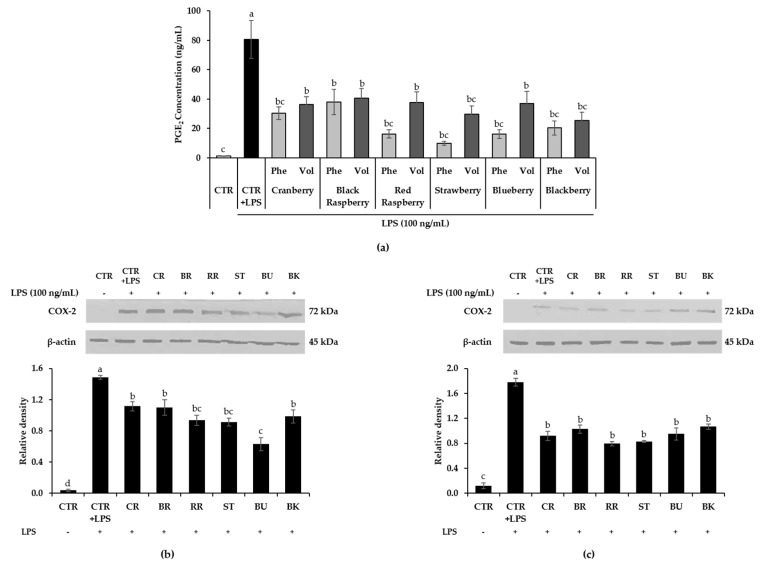
(**a**) Effect of berry phenolic and volatile extracts (50-fold dilution) on LPS-induced PGE_2_ production in RAW264.7 macrophages. Effect of (**b**) berry phenolics and (**c**) berry volatiles (50-fold dilution) on LPS-stimulated COX-2 production in RAW264.7 cells. Data are expressed as the mean ± SEM (*n* = 4). Means not sharing any letter are significantly different by the Tukey’s test at the 5% level of significance. CTR = control; CR = cranberry; BR = black raspberry; RR = red raspberry; ST = strawberry; BU = blueberry; BK = blackberry.

To investigate the effect of six berry phenolic and volatile extracts on LPS-stimulated inflammatory mediators, PGE_2_ and COX-2 production were detected by ELISA and Western blot analysis, respectively (Figure 3). Compared to the control, LPS-stimulated control excessively produced PGE_2_ level (*p* < 0.01). All six berry phenolic and volatile extracts significantly inhibited PGE_2_ production in LPS-induced RAW264.7 cells compared to the LPS-treated control (*p* < 0.05). Each berry phenolic extract showed a stronger inhibitory effect on LPS-induced PGE_2_ production than its volatile extract, but there was no significant difference between two extracts of each berry. In Figure 3b,c, the LPS-treated control group significantly produced COX-2 in RAW264.7 cells (*p* < 0.01). COX-2 production in LPS-treated RAW264.7 cells was significantly suppressed by both phenolic and volatile extracts from all six berries, decreasing 25–58% and 40–55%, respectively (*p* < 0.05). All berry volatile extracts exerted comparable suppression on COX-2 production to respective berry phenolic extracts, showing no significant difference.

### 3.7. Effect of Berry Phenolic and Volatile Extracts on LPS-Induced Pro-Inflammatory Cytokines Production

**Figure 4:**
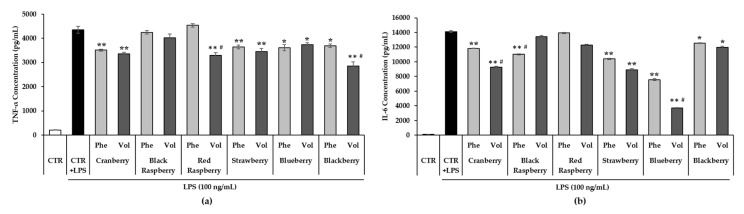
Effect of berry phenolic and volatile extracts (50-fold dilution) on (**a**) TNF-α and (**b**) IL-6 production. Data are expressed as the mean ± SEM (*n* = 5). The significant difference was compared with the LPS-stimulated control at * *p* < 0.05, ** *p* < 0.01. # indicates a significant difference compared between phenolic and volatile treatment within each berry (*p* < 0.05). CTR = control; Phe = phenolic extract; Vol = volatile extract.

To further analyze the anti-inflammatory effect of six berry phenolic and volatile extracts, the production of LPS-induced pro-inflammatory cytokines involving TNF-α and IL-6 in the cultured medium was determined by ELISA (Figure 4). The LPS-treated control group showed a significant increase in the TNF-α level compared to the control without LPS by more than 20 times (*p* < 0.01). However, all 50-fold diluted berry phenolic and volatile extract treatments noticeably suppressed the TNF-α concentration in LPS-stimulated RAW264.7 cells (*p* < 0.05) except black raspberry phenolic and volatile extract and red raspberry phenolic extract. Furthermore, all six berry volatile extract treatments reduced the level of TNF-α comparably or more significantly than the respective berry phenolic extract treatments. The control group stimulated with LPS significantly induced the level of IL-6 compared to the control without LPS, which barely produced IL-6 by more than 20 times (*p* < 0.01). However, all berry phenolic and volatile extract treatments showed a significant decrease in IL-6 concentration on LPS-stimulated RAW264.7 cells (*p* < 0.05) except black raspberry volatile and red raspberry phenolic and volatile extract treatments. Except black raspberry, all six berry volatile extract treatments exhibited a comparable or stronger suppression effect on LPS-induced IL-6 production than the respective berry phenolic extract treatments.

### 3.8. Effect of Berry Phenolic and Volatile Extracts on LPS-Stimulated NF-κB Activation

**Figure 5:**
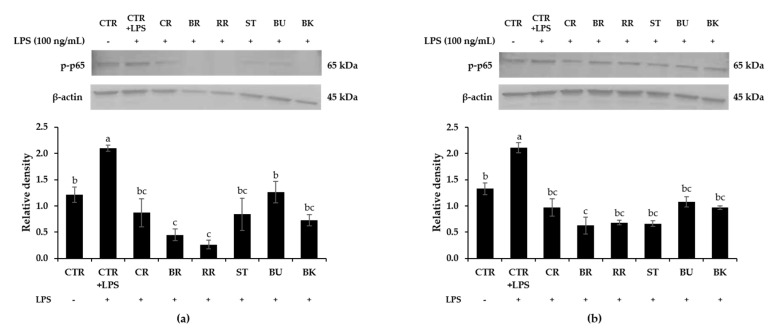
Inhibitory effect of berry (**a**) phenolic and (**b**) volatile extracts (50-fold dilution) on LPS-induced phosphorylation of NF-κB p65 in RAW264.7 macrophages. Means not sharing any letter are significantly different by the Tukey’s test at the 5% level of significance. CTR = control; CR = cranberry; BR = black raspberry; RR = red raspberry; ST = strawberry; BU = blueberry; BK = blackberry.

**Figure 6:**
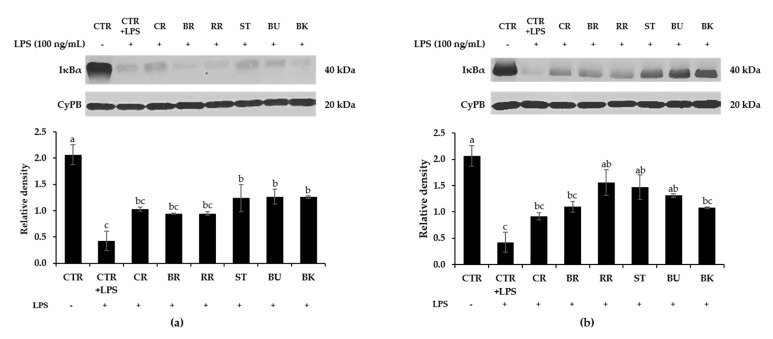
Inhibitory effect of berry (**a**) phenolic and (**b**) volatile extracts (50-fold dilution) on LPS-induced phosphorylation of IκBα in RAW264.7 macrophages. Means not sharing any letter are significantly different by the Tukey’s test at the 5% level of significance. CTR = control; CR = cranberry; BR = black raspberry; RR = red raspberry; ST = strawberry; BU = blueberry; BK = blackberry.

To further investigate the pathway of the anti-inflammatory effect, the effect of berry phenolic and volatile extracts on LPS-stimulated NF-κB activation was evaluated by Western blot (Figure 5). The level of phosphorylated p65 (p-p65) on LPS-stimulated RAW264.7 treated with berry phenolic and volatile extracts was measured. The phosphorylation of p65 was significantly escalated by LPS treatment alone compared to the control without LPS (*p* < 0.05). Compared to the control with LPS group, all six berry phenolic and volatile extracts significantly inhibited the level of p-p65 (*p* < 0.05). Each berry volatile extract showed a comparable inhibitory effect on the p65 phosphorylation to the respective phenolic extracts. Among berry phenolic extracts, red raspberry and black raspberry showed higher inhibition compared to the others, and black raspberry showed the highest suppression among the berry volatile extracts. In addition, the degradation of IκBα (inhibitor protein of κBα) on LPS-induced RAW264.7 with berry phenolic and volatile extract treatments is shown in Figure 6. Compared to the control without LPS treatment, LPS treatment alone remarkably decreased the level of IκBα, showing a significant degradation of IκBα (*p* < 0.05). All berry phenolic and volatile extract treatments showed less degradation of IκBα than the control treated with LPS. Among berry phenolics, strawberry, blueberry and blackberry exerted stronger inhibition on IκBα degradation and red raspberry, strawberry and blueberry reduced less amount of IκBα among berry volatile extract treatments, showing significant difference to LPS-treated control group (*p* < 0.05). These results showed that berry phenolic and volatile extracts inhibited the activation of inflammatory transcription factor NF-κB by blocking the phosphorylation of p65 (p-p65) and degradation of the inhibitor protein of NF- κB (IκBα).

## 4. Discussion

Berries, one of the most commonly consumed fruits in the human diet, have received much attention because of their nutritive (vitamins and minerals) and non-nutritive compounds such as polyphenols and volatiles [77,78]. However, there is not much information available on the composition and health-promoting effect of volatiles in berries, to the best of knowledge. Therefore, we analyzed the composition of berry phenolic and volatile fractions and investigated the antioxidant and anti-inflammatory activities of berry volatile extracts, comparing the results to berry phenolic extracts.

The biological properties including antioxidant, antimicrobial, anti-inflammatory and anticancer have been attributed to the diverse array of phenolic compounds present in berries, including flavonoids (anthocyanins, flavonols and flavanols), stilbenoids (resveratrol, pterostilbene, piceatannol), tannins (proanthocyanidins and ellagitannins) and phenolic acids (hydroxybenzoic acid and hydroxycinnamic acids) [79]. In this study, we found that black raspberry phenolic extract had the highest total phenolic and anthocyanin contents, while strawberry has the lowest total phenolic and anthocyanin contents among the six berries tested. Differences in total phenolic and total anthocyanins between some of our values and previous reported values are likely due to differences in genetics, extraction and HPLC methods.

Bioavailability of berry phenolics in vivo is usually very low in many studies [80,81,82,83]. Anthocyanins are one of the most abundant polyphenols in berries, but its bioavailability ranged from less than 0.1% to 2% from ingested anthocyanin [84]. There are not many studies that have evaluated the bioavailability of berry volatiles after oral administration, but studies showed that volatiles are quickly absorbed and eliminated [85]. From these findings, we decided to treat very low amounts of berry phenolics and volatiles on RAW264.7 cells for 1 h. Berry phenolics and volatiles extracted in this study are a mixture of a wide range of different individual phenolic and volatile compounds, respectively, with each berry having a different composition. Thus, we decided to use dilutions (50, 100 and 200-fold dilution) of the fresh berry phenolic and volatile extracts to directly compare the bioactivities of phenolic and volatile extracts. With the 50× dilution of the berry extracts used in this study, the RAW264.7 cells were exposed to low concentrations of phenolics ranging from 26 μg/g for strawberries to 237 μg/g for black raspberries. Conversely, the comparable or higher anti-inflammatory activity of the volatiles was remarkable, considering the 50× dilution and the very low concentrations of volatiles ranging from 29 ng/g for blueberries to 244 ng/g for blackberries. Our results indicate that volatile compounds possess potent anti-inflammatory activity at very low concentrations.

Macrophages play an important role in the immune system as the host defense [86]. When they are exposed to LPS/ foreign particles or injury, they excessively induce pro-inflammatory mediators (NO, PGE_2_ and COX-2) and cytokines such as TNF-α and IL-6 [87]. Excessive produced NO by LPS stimulation can yield DNA damage, induction of apoptosis and oxidative stress and low-density lipoprotein (LDL) oxidation, increasing inflammation [88,89]. Black and red raspberry crude extracts (100, 150 and 200 μg/mL) showed a remarkable reduction in the LPS/IFN-γ-induced NO level by 20%–25% [90]. In the study of Cheng et al. [91], they prepared two types of blueberry polyphenols for investigating the anti-inflammatory effect on LPS-induced RAW264.7 cells, soluble (extractable) polyphenols from cell vacuoles in plant and insoluble (nonextractable) polyphenols from the plant cell wall. Insoluble polyphenols contain dietary fiber-bound hydrolysable tannins and proanthocyanidins. The NO level treated with extractable and nonextractable blueberry polyphenol extracts (10, 100, 200 and 400 μg/mL) showed a dose-dependent decrease in LPS-stimulated RAW264.7. Strawberry extract (100 μg/mL) also a showed significant decrease in LPS-stimulated NO production, compared to the control without LPS (*p* < 0.05) [92]. In a recent study, Moore et al. [28] compared the NO-inhibitory effect of cranberry polyphenols and volatiles. Both cranberry polyphenol (317.8 and 635.7 μg/g) and volatile (1.8 μg/g) extracts significantly suppressed LPS-induced NO production (*p* < 0.05). Similarly, our results showed that all six berry phenolic or volatile extracts notably suppressed LPS-induced NO production (Figure 2). Interestingly, blueberry exhibited the highest inhibitory effect, followed by strawberry, red raspberry and blackberry. Our results demonstrated that berry phenolic and volatile extracts at the tested dilution had no cytotoxic effect, suggesting that the inhibitory effect on LPS-stimulated NO level was not associated with their cell cytotoxicity (Figure 1). 

PGE_2_ is one of inflammatory mediators produced by COX-2 in inflammatory sites [93]. Cyclooxygenase is an enzyme involved in prostaglandin synthesis, converting arachidonic acid to prostaglandins [94]. Isoenzyme COX-1 normally exists to regulate physiological activities in most cells, regardless of stimuli. However, another isoenzyme, COX-2, is highly expressed in inflammatory conditions as an inducible enzyme by endogenous and exogenous stimuli such as cytokines, oxidative stress and LPS [95]. When COX-2 is excessively expressed, it can induce inflammation, tumor progression and metastasis, developing cancer, cardiovascular diseases and rheumatoid arthritis [96,97]. Thus, targeting the inhibition of COX-2 and PGE_2_ is believed to be an important factor for resolving the inflammatory mechanism [98]. Blackberry extracts (50 μg/mL) from the ”Jumbo” and ”Black Satin” cultivar remarkably decreased COX-2 gene expression by more than 30% compared to the LPS-treated control [99]. Anthocyanin-rich fractions from red raspberry (150 μg/mL) significantly reduced both mRNA and protein levels of COX-2 [90]. COX-2 mRNA expression was suppressed in a concentration-dependent manner in LPS-stimulated RAW264.7 cells with the treatment of extractable and nonextractable polyphenol extracts of blueberry, decreasing over 60% and 40%, respectively [91]. The root and unripe fruit polyphenols of black raspberry showed a concentration-dependent decrease in LPS-induced PGE_2_ secretion in a 1–100 μg/mL concentration range [100]. The present study found that LPS-stimulated PGE_2_ production was highly diminished by all six berry phenolic and volatile extract treatments (*p* < 0.05). Especially, all berry phenolics except black raspberry exerted no significant differences in PGE2 production level relative to control without LPS. Both phenolics and volatiles treatment of all six berries significantly attenuated LPS-stimulated COX-2 level in RAW 264.7 cells (*p* < 0.05).

TNF-α accelerates other pro-inflammatory cytokines and gene expression, inducing the risk of rheumatoid arthritis, asthma and inflammatory bowel disease [101,102,103]. IL-6 is one of the main cytokines related to developing inflammatory and autoimmune diseases such as rheumatoid arthritis, systemic sclerosis, systemic lupus erythematosus, asthma and other diseases [104,105,106,107]. Therefore, suppressing the pro-inflammatory cytokines may be a part of a therapeutic treatment for preventing or treating inflammatory related diseases. Su et al. [108] tested 16 different blueberry samples from China and reported that samples containing higher phenolic acid amounts showed a higher suppressing effect on TNF-α and IL-6. Black Satin and Jumbo cultivar blackberry extracts (50 and 100 mg/mL) slightly suppressed the NO level, but significantly inhibited gene expression of IL-6 [99]. Lean C57BL/6 mice fed dietary powder of strawberry showed down-regulation of IL-6, IL-1β and TNF-α [109]. Our results exhibited that all berry phenolic and volatile extracts except black raspberry and red raspberry significantly lowered the level of TNF-α and IL-6 in LPS-activated RAW264.7 cells. Consistent with our findings, Kim et al. [100] showed that black raspberry polyphenols (1, 5, 10, 25, 50 and 100 μg/mL) significantly inhibited LPS-induced NO and IL-6 production, but slightly suppressed the TNF-α level with no significance. Anthocyanin-rich fractions from red raspberry (150 and 200 μg/mL) remarkably reduced IL-6 mRNA expression [90]. In addition, significant TNF-α and IL-6 inhibition was exhibited with the treatment of cyanidins from black raspberry [110]. Both studies using individually purified phenolic compounds demonstrated a noticeable inhibitory effect, suggesting that more purified or fractionated individual phenolic compounds from black raspberry and red raspberry with higher concentrations may exhibit a stronger anti-inflammatory effect on pro-inflammatory cytokines.

NF-κB is a transcription factor that regulates the gene expression of inflammation, cell growth, proliferation and immune response [111]. NF-Κb is composed of two subunits—p65 and p50. These two subunits are inactivated in cytosol by binding to the inhibitor protein of κBα (IκBα). However, if LPS, TNF-α, or other pro-inflammatory cytokines/mediators stimulate inactivated NF-κB, IκBα is phosphorylated and degraded [112]. It results in the phosphorylation of p65 (p-p65) and activation of p65 transcription activity after translocating into the nucleus [113]. Li et al. [90] showed that anthocyanin-rich fractions of red raspberry polyphenols significantly suppressed the activation of NF-κB by inhibiting the phosphorylation of p65 and IκBα. In this study, all six berry phenolic and volatile extracts noticeably inhibited the activation of inflammatory transcription factor NF-κB by blocking the phosphorylation of p65 (p-p65) and degradation of the inhibitor protein of NF-κB (IκBα). Collectively, our findings showed that all six berry phenolic or volatile extracts significantly inhibited the production of NO, TNF-α and IL-6 through suppression of the NF-κB signaling pathway, suggesting that six berry phenolics and volatiles may have a potential anti-inflammatory effect on inflammatory related diseases.

In the present study, monoterpenes were the most abundant volatiles in all berry volatile extracts except blackberry, where acids predominated. Monoterpenes are secondary metabolites produced by plants, and they possess many health beneficial effects [114]. Among monoterpenes, myrtenol, which was the major volatile compound in strawberry, red raspberry and black raspberry volatile extracts, showed anti-inflammatory and antinociceptive effects in mice through suppressing inflammatory mediators and the signaling pathway of receptors related to pain transmission [115]. Viana et al. [116] tested the gastroprotective effect of (−)-myrtenol from gastric lesions caused by ethanol in mice and found that (−)-myrtenol significantly exhibited gastroprotection against acute gastric lesions. Linalool, which is one of the main components in berry volatile fractions, significantly down-regulated the phosphorylation of IκBα in LPS-activated RAW264.7 cells [117]. Furthermore, α-terpineol showed antitumor activity in different tumor cell lines, especially in the small cell lung carcinoma cell line, by suppressing the signaling pathway of NF-κB [118]. These studies, reporting many health-promoting effects including anti-inflammatory activities, suggest that the anti-inflammatory effect of berry volatile extracts in this study might be due to monoterpenes—the predominant class of volatiles present in berries. Further research is needed to determine specific volatile compounds responsible for anti-inflammatory activity.

## 5. Conclusions

According to our results, cranberry, black raspberry, red raspberry, strawberry, blueberry and blackberry contain a diverse composition of volatile compounds, especially monoterpenes predominant in all berries but blackberries. Although present at a much lower concentration than phenolics, most of the berry volatile extracts exerted a comparable or higher anti-inflammatory effect than berry phenolic extracts through suppression of pro-inflammatory cytokines and mediators via down-regulation of the NF-κB signaling pathway. In conclusion, the present study suggests that volatiles in six berries may have the potential for anti-inflammatory activity to prevent many inflammatory diseases.

## Figures and Tables

**Table 1:** Phytochemical composition of berry phenolic and volatile extracts.

Berry	Phenolic Extract			Total Volatiles	Volatile Extract
	TPH ^1^ (mg GAE/100 g)	TACY ^2^ (mg/100 g)	DPPH (µM TE/100 g)	(µg/kg)	DPPH (µM TE/kg)
Cranberry	421.3 b ^3^	98.6 c	1806.5 bc	2864.9 c	4.93 a
Black raspberry	1186.4 a	342.0 a	2761.8 a	8556.1 b	7.73 a
Red raspberry	208.1 c	19.1 e	1474.8 d	2055.5 cd	6.57 a
Strawberry	125.4 d	8.7 e	643.6 e	2989.7 c	8.62 a
Blueberry	389.5 b	184.9 b	1674.8 c	1424.1 d	ND
Blackberry	386.2 b	70.2 d	1980.2 b	12,212.9 a	ND

^1^ Total phenolics (TPH) measured by the Folin–Ciocalteu assay. ^2^ Total anthocyanins (TACY) measured by the pH differential assay. ^3^ Values represent means (*n* = 3). Values within columns with different letters are significantly different (*p* < 0.05).

**Table 2:** The class composition of berry volatile extracts (µg/kg).

Class	Cranberry	Black Raspberry	Red Raspberry	Strawberry	Blueberry	Blackberry
Monoterpene	1727.1 ± 129.3 ^1^	5217.6 ± 562.9	958.7 ± 240.3	1293.9 ± 144	639.6 ± 129.9	1163.1 ± 150.6
Alcohol	176.6 ± 50.6	1163.5 ± 183.3	89.9 ± 27.5	85.4 ± 22.9	247.1 ± 96	2198.1 ± 121.4
Aldehyde	158.6 ± 23.4	647 ± 315.8	106.4 ± 9.8	158.9 ± 29.9	110.2 ± 31.3	39.4 ± 8.1
Ester	413.6 ± 46.3	836.6 ± 189	42.5 ± 9.4	414.4 ± 108.6	93.8 ± 27.8	1240.8 ± 201.4
Ketone	50.3 ± 2.6	78 ± 6.2	52.9 ± 14.7	84.1 ± 24.6	62.8 ± 13.4	159.9 ± 13.5
Acid	338.7 ± 39.4	312.9 ± 51.4	489.7 ± 231	597.1 ± 251.9	33.4 ± 15	6967.2 ± 503.7
C13 norisoprenoid		126 ± 30.5	186.3 ± 33.5	11.7 ± 1.1	96.2 ± 9.2	90.8 ± 61.4
Sesquiterpene		43.4 ± 11.2			8.6 ± 2.2	
Phenolic		32.3 ± 12	78.8 ± 8.7	10.6 ± 2.3	32 ± 0.8	4.6 ± 4.6
Lactone		24.4 ± 3.9	30.9 ± 3.8	8.8 ± 1.1		55.1 ± 27
Alkylbenzene		34.7 ± 5.1	4.4 ± 1.1	99.5 ± 13.8		
Furan		38.5 ± 19.3		206.8 ± 34.8	67 ± 26.3	293.9 ± 38.5
Furanone			8.9 ± 4.3		33.4 ± 11.2	
Hydrocarbon		1.2 ± 1.2	6.1 ± 1.4	11.8 ± 3.0		
Benzothiazole				6.7 ± 0.9		

^1^ Values represent means (*n* = 3) ± SEM.

## Supplementary Materials

The supplementary materials are available online at https://www.mdpi.com/2076-3921/9/9/871/s1.
