# Supplementary material for: Berry Phenolic and Volatile Extracts Inhibit Pro-Inflammatory Cytokine Secretion in LPS-Stimulated RAW264.7 Cells through Suppression of NF-κB Signaling Pathway
Source: Antioxidants (Basel). 2020 Sep 15;9(9):871. doi: 10.3390/antiox9090871 (PMC7554842; doi:10.3390/antiox9090871)
Supplement: Supplementary file 1 [file antioxidants-09-00871-s001.pdf]

Table S1. Polyphenol content (mg/100 g) of cranberry extract

Anthocyanins		Flavonols		Procyanindins		Phenolic acids	
Cyanidin-3-galactoside	15.9±0.4[1]	Myrcetin-3-pentoside	0.7±0.1	Procyanidin dimers	0.4±0.0	Chlorogenic acid	18.4±0.3
Cyanidin-3-glucoside	0.7±0.0	Quercetin-3-xyloside	0.4±0.1	Procyanidin trimer	1.0±0.1		
Cyanidin-3-arabinoside	9.3±0.3	Quercetin-3-arbinopyranoside	1.4±0.2				
Malvidin-3-galactoside	1.3±0.2	Myrcetin-3-galactoside	1.6±0.2				
Peonidin-3-galactoside	46.3±0.4	Quercetin-3-arabinofuranoside	0.3±0.3				
Peonidin-3-glucoside	4.4±0.2	Quercetin-3-galactoside	3.5±0.4				
Peonidin-3-arabinoside	18.6±0.5	Quercetin-3-glucoside	0.3±0.3				
		Quercetin-3-rhamnoside	0.4±0.4				
		Isorhamnetin-3-hexoside	0.4±0.4				
		Quercetin-3-coumaroyl-galactoside	0.1±0.1				
		Isorhamnetin-3-pentoside	0.2±0.2				
		Isorhamnetin-3-pentoside	0.3±0.3				
		Isorhamnetin-3-pentoside	0.1±0.1				
		Isorhamnetin-3-pentoside	0.2±0.2				
		Syringetin-3-pentoside	0.1±0.1				
		Quercetin-3-benzoyl-galactoside	0.1±0.1				
Total anthocyanins	96.5±0.4	Total flavonols	10.1±0.5	Total procyanidins	1.4±0.1	Total phenolic acids	18.4±0.3

[1]Values represent means (n=3) ± SEM.

Table S2. Polyphenol content (mg/100 g) of black raspberry extract

[1]Values represent means (n=3) ± SEM.

**Anthocyanins**		**Flavonols**		**Ellagitannins**	
Cyanidin-3-sophoroside-5-rhamnoside	3.5±0.3[1]	Quercetin-3-rutin	10.0±0.1	Castalagin/vescalagin isomer	3.8±0.1
Cyanidin-3-glucoside	117.3±1.7	Quercetin-3-glucuronide	11.8±0.2	Castalagin/vescalagin isomer	3.3±0.1
Cyanidin-3-rutinoside	249.3±2.5	Quercetin-3-glucoside	5.6±0.4	Pedunculagin (bis-HHDP-glucose) isomers	2.9±0.0
Cyanidin-3-sambubioside-5-rhamnoside	91.6±0.5	Quercetin derivative	2.8±0.2	Pedunculagin (bis-HHDP-glucose) isomers	0.7±0.0
Pelargonidin-3-rutinoside	22.3±0.7	Isorhamnetin-3-pentoside	1.6±0.0	Galloyl-HHDP-glucose	0.8±0.0
		Isorhamnetin derivative	2.9±0.1	Sanguiin H-10 (isomer)	1.4±0.1
				Sanguiin H-10 (isomer)	5.8±0.1
				Sanguiin H-6/ Lambertianin A (isomer)	4.7±0.2
				Sanguiin H-6/ Lambertianin A (isomer)	12.5±0.5
				Lambertianin C	4.5±0.1
				Acetyl-methyl-EA-pentoside	0.8±0.0
Total anthocyanins	484.0±5.3	Total flavonols	34.7±0.3	Total ellagitannins	42.4±0.4

Table S3. Polyphenol content (mg/100 g) of red raspberry extract

**Anthocyanins**		**Flavonols**		**Ellagitannins**		**Procyanindins**	
Cyanidin-3-sophoroside	5.1±0.4[1]	Kaempherol-3-rutinoside-7-rhamnoside	0.3±0.1	Sanguiin H-6 without gallic moiety	0.4±0.1	Procyanidin dimers	6.6±0.5
Cyanidin-3-sophoroside-5-rhamnoside	10.8±0.6	Quercetin-3-rutinoside	1.6±0.2	Sanguiin H-10	1.0±0.1	Procyanidin trimer	1.7±0.2
Cyanidin-3-glucoside	5.1±0.3	Quercetin-3-glucuronide	1.2±0.1	Galloyl-bis-HHDP-glucose isomers	0.2±0.0		
Cyanidin-3-rutinoside	10.0±0.5			Sanguiin H-6/ Lambertianin A (isomer)	0.6±0.0		
Pelargonidin-3-rutinoside	0.9±0.0			Sanguiin H-6/ Lambertianin A (isomer)	8.3±0.3		
				Sanguiin H-2	0.3±0.0		
				Lambertianin C	4.0±0.2		
Total anthocyanins	32.0±0.4	Total flavonols	3.1±0.1	Total ellagitannins	14.6±0.2	Total procyanidins	8.3±0.7

[1]Values represent means (n=3) ± SEM.

Table S4. Polyphenol content (mg/100 g) of strawberry extract

**Anthocyanins**		**Flavonols**		**Ellagitannins**	
Pelargonidin-glucoside	17.8±0.4	Quercetin-3-glucuronide	1.9±0.2	Castalagin/vescalagin isomer	1.3±0.1
Pelargonidin-3-rutinoside	1.3±0.1	Quercetin derivative	0.7±0.0	Pedunculagin (bis-HHDP-glucose)	0.1±0.0
Pelargonidin-3-malonoyl-glucoside	3.4±0.2	Quercetin derivative	0.9±0.0		
		Kaempferol-3-hexoside	0.4±0.0	Pedunculagin (bis-HHDP-glucose) isomers	0.4±0.0
		Kaempferol derivative	0.9±0.1	Galloyl-HHDP-glucose	0.2±0.0
		Kaempferol derivative	0.2±0.0	Galloyl-bis-HHDP-glucose isomers	0.0±0.0
		Kaempferol-3-rutinoside	0.1±0.0	Sanguiin H-6/Lambertianin A	0.2±0.0
				Lambertianin C	1.0±0.0
				Unknown	0.2±0.0
				Methyl-EA pentosides	0.1±0.0
Total anthocyanins	22.6±0.4	Total flavonols	5.1±0.2	Total ellagitannins	3.5±0.1

[1]Mean values (n=3) ± SEM.

Table S5. Polyphenol content (mg/100 g) of blueberry extract

[1]Mean values (n=3) ±SEM.

Anthocyanins		Flavonols		Procyanidins		Phenolic acids	
Delphidin-3-galactoside	41.9±0.4[1]	Myrcetin-3-rhamnoside	0.8±0.0	Procyanidin dimers	1.2±0.1	Chlorogenic acid	21.2±0.3
Delphinidin-3-glucoside	1.5±0.1	Quercetin-3-rutinoside	2.4±0.1	Procyanidin trimer	2.4±0.1		
Delphinidin-3-arabinoside	15.7±0.2	Isorhamnetin-7-deoxyhexoside	0.5±0.1				
Cyanidin-3-galactoside	15.3±0.2	Quercetin-3-glucuronide	4.9±0.4				
Cyanidin-3-glucoside	1.1±0.0	Quercetin-3-galactoside	3.8±0.2				
Cyanidin-3-arabinoside	5.1±0.1	Quercetin-3-glucoside	2.2±0.1				
Petunidin-3-galactoside	23.8±0.3	Quercetin-3-pentoside	2.0±0.1				
Petunidin3-glucoside	1.4±0.1	Quercetin-3-rhamnoside	3.2±0.2				
Petunidin-3-arabinoside	8.2±0.2	Syringetin-3-glucoside	0.4±0.0				
Malvidin-3-galactoside	45.5±0.5	Syringetin-3-glucuronide	0.5±0.0				
Malvidin-3-glucoside	4.8±0.1	Syringetin-3-rhamnoside	0.2±0.0				
Malvidin-3-arabinoside	15.4±0.3						
Peonidin-3-galactoside	10.6±0.2						
Total anthocyanins	190.4±0.5	Total flavonols	20.9±0.4	Total procyanidins	3.6±0.1	Total phenolic acids	21.2±0.3

Table S6. Polyphenol content (mg/100 g) of blackberry extract

[1]Values represent means (n=3) ± SEM.

**Anthocyanins**		**Flavonols**		**Ellagitannins**		**Procyanindins**	
Cyanidin-3-glucoside	91.2±0.4[1]	Quercetin-3-(3-hydroxy-methylglutaroyl-galactoside)	1.6±0.1	Pedunculagin (bis-HHDP-glucose) isomers	0.3±0.0	Procyanidin dimers	3.4±0.2
Cyanidin-3-xyloside	4.1±0.1	Quercetin-3-oxalyl-pentoside	0.6±0.0	Pedunculagin (bis-HHDP-glucose) isomers	0.0±0.0	Procyanidin trimers	3.6±0.1
Cyanidin-3-malonyl-glucoside	2.3±0.1	Quercetin-3-rutinoside	1.7±0.1	Pedunculagin (bis-HHDP-glucose) isomers	0.1±0.0		
Cyanidin-3-dioxalyl-glucoside	1.0±0.0	Isorhamnetin derivative	2.5±0.3	Pedunculagin (bis-HHDP-glucose) isomers	0.1±0.0		
Malvidin derivative	0.6±0.0	Quercetin derivative	2.4±0.2	Galloyl-HHDP-glucose	0.2±0.0		
		Quercetin-3-glucuronide	1.8±0.1	Sanguiin H-10	1.1±0.1		
		Quercetin-3-galactoside	1.4±0.0	Galloyl-bis-HHDP-glucose isomer	0.2±0.0		
		Quercetin-3-malonyl-gluccoside	2.4±0.3	Galloyl-bis-HHDP-glucose isomer	1.4±0.1		
		Kaempherol-3-hexoside	0.8±0.0	Sanguiin H-6/Lambertianin A (isomer)	2.0±0.2		
				Sanguiin H-6/Lambertianin A (isomer)	3.2±0.3		
				Sanguiin H-2	0.2±0.0		
				Lambertianin C	7.2±0.4		
Total anthocyanins	99.1±0.4	Total flavonols	15.2±0.3	Total ellagitannins	16.0±0.3	Total procyanidins	7.0±0.2

Table S7. Retention indices (RI), identification, classification and concentration of volatiles (μg/kg) in cranberry, black raspberry, strawberry, blackberry, red raspberry and blueberry extracts

RI	Volatile	Class	Cranberry	Black Raspberry	Red Raspberry	Strawberry	Blueberry	Blackberry
586	Diacetyl (Butan-2,4-dione)	Ketone				40.7±14.4[1]		
619	Ethyl acetate	Ester		340.1±14.7				
725	Isoamyl alcohol	Alcohol				15.5±9.5		
774	2-Buten-1-ol, 3-methyl-	Alcohol		40.9±4.8				
800	Hexanal	Aldehyde	53.3±5.0		11.3±1.9	46.7±4.6	38.3±1.0	
804	Ethyl butanoate	Ester				200.0±13.4		
820	Butanoic acid	Acid			311.5±34.3	209.7±61.4		4732.3±468.8
827	Furfural	Furan					67.0±26.3	241.8±27.8
839	4-Methyl-1-pentanol	Alcohol						1230.6±50.8
850	Hexanol	Alcohol	10.7±8.4				10.5±0.5	
855	Ethyl 3-methylbutanoate	Ester					8.5±8.5	
859	(Z)-3-hexen-l-ol cis	Alcohol						160.3±2.4
880	2-Hexen-1-ol (Z) cis	Alcohol						41.9±0.6
880	3-Methylbutanoic acid	Acid			97.5±119.4			
888	2,5-Dimethyl-5-hexen-3-ol	Alcohol		69.8±18.9				
890	2-Methylbutyric acid	Acid	338.7±39.4		60.2±73.7			
896	Styrene (ethenyl benzene)	Hydrocarbon		1.2±1.2		11.8±3.0		
900	2-Heptanone	Ketone			0.5±0.7		40.1±0.7	11.4±3.4
904	Heptanal	Aldehyde	6.9±3.5	29.1±29.1		47.4±18.2		
913	Pentanoic acid	Acid					23.3±12.3	
917	Ethyl-3-hydroxybutanoate	Ester		181.7±129.0				631.7±152.2
926	2-Heptanol	Alcohol		7.5±2.5	17.2±8.0	24.2±4.0		20.4±1.4
930	2-Heptanol	Alcohol					19.4±12.3	9.7±0.0
939	α-Thujene	Monoterpene						8.2±1.3
943	α-Pinene	Monoterpene		3.8±0.5		8.2±1.1	7.9±1.3	
952	Camphene	Monoterpene		10.6±5.3				14.9±1.0

957	Benzaldehyde	Aldehyde	4.4±0.6	9.4±5.2			5.2±5.2	
961	2-Ethyl-hexanal	Aldehyde					27.8±11.2	
965	1-Heptanol	Alcohol		158.5±31.7				67.5±3.9
970	β-Thujene	Monoterpene	3.2±0.7					14.1±5.9
970	Sabinene	Monoterpene		3.9±3.9				
974	1-Octen-3-one	Ketone					22.7±12.7	
978	2-Menthene	Monoterpene		14.9±2.0				
983	β-pinene	Monoterpene	16.5±5.6	43.3±5.1		31.1±3.4		
983	1-Octen-3-ol	Alcohol	23.0±12.4		19.6±5.5		15.1±9.3	89.3±9.8
987	6-Methyl-5-hepten-one (sulcutone)	Ketone	40.2±1.5					
987	α-myrcene	Monoterpene			4.4±5.4			
992	β-myrcene	Monoterpene	12.1±1.8	66.9±2.3	9.9±1.9		6.4±1.2	8.7±3.7
996	6-Hepten-1-ol, 2-methyl-	Alcohol	6.8±2.0		7.3±4.9			
996	(E)-2-Hexenyl acetate	Ester					21.5±0.7	
1000	Methyl hexanoate	Ester			19.3±2.6	15.3±7.7		
1000	Ethyl hexanoate	Ester	130.1±14.7			10.9±1.1		498.4±38.2
1000	β-Pyronene	Monoterpene		26.3±2.0				
1005	Octanal	Aldehyde	32.2±3.8	12.4±6.4				
1005	α-Phellandrene	Monoterpene			7.9±0.9			
1010	(Z)-3-Hexenyl acetate	Ester					36.4±2.3	
1014	α-Terpinene	Monoterpene	11.3±0.8	67.0±5.5	2.0±1.2			
1014	Hexyl acetate	Ester				2.2±1.1		
1024	Hexanoic acid	Acid		312.9±51.4				2234.9±34.9
1024	3-Ethyl-4-methylpentanol	Alcohol						102.6±8.8
1029	Eucalyptol	Monoterpene	336.8±45.2					
1029	p-Cymene	Monoterpene		62.1±10.4				75.6±4.4
1033	Limonene	Monoterpene	25.2±3.3		1.8±0.4		4.2±0.6	164.7±5.9
1033	2-Ethylhexanol	Alcohol		618.3±99.8			82.2±0.4	290.2±25.7
1033	Methyl 3-hydroxyhexanoate	Ester				2.7±1.4		
1038	Benzyl alcohol	Alcohol	50.9±7.6	146.1±14.7	2.2±0.2	4.5±2.4	3.0±0.5	

1043	Acetophenone	Ketone	10.1±1.1			1.9±1.0		
1043	o-Cymene	Monoterpene	13.0±0.7					
1043	Octanoic acid, methyl ester	Ester		64.3±11.0				
1048	Phenylacetaldehyde	Aldehyde					3.6±1.4	
1052	ß-Phellandrene	Monoterpene		64.7±3.7				
1052	Dihydromyrcenol	Monoterpene				20.2±4.2		
1062	Mesifuran	Furan				206.8±34.8		12.9±1.9
1062	Furaneol	Furanone			8.1±3.4		33.4±11.2	
1067	(Z)-Linalool oxide, cis	Monoterpene		69.1±7.8	15.0±6.8		37.4±20.9	10.0±0.5
1067	Dihydromyrcenol	Monoterpene	17.3±1.9					
1071	Octanol	Alcohol	13.2±2.5			18.0±1.7		
1076	γ-Terpinene	Monoterpene	5.5±2.7					
1081	Linalool oxide (pyran)	Monoterpene		7.8±0.4				16.2±2.4
1081	2,3-Butanediol, diacetate	Ester				18.7±3.7		
1086	m-Cresol	Phenolic		13.2±3.9				
1086	Non-1-en-3-ol	Alcohol				16.1±3.7		
1090	p-α-Dimethyl styrene	Alkylbenzene		34.7±5.1	4.4±1.1	99.5±13.8		
1090	α-Terpinolene	Monoterpene			3.9±2.8		13.9±10.8	10.5±2.2
1095	2-Nonanone	Ketone						3.4±3.4
1100	Linalool	Monoterpene	210.0±19.4	1138.4±65.8	140.4±2.4	28.8±3.7	186.9±15.5	10.6±0.1
1105	Nonanal	Aldehyde	31.0±6.4	61.9±1.4		46.4±3.9		2.4±2.4
1111	Dihydrolinalool oxide	Monoterpene					28.4±22.8	
1111	Ethyl 3-acetoxybutyrate	Ester				15.6±7.8		
1121	Isophorone	Ketone				11.5±1.4		139.0±0.6
1121	Phenylethyl alcohol	Alcohol	32.8±6.7	72.9±1.5	5.8±1.2		111.2±71.4	138.0±7.5
1126	Allo-ocimene	Monoterpene		16.6±4.0			29.8±18.6	
1132	Limonene oxide	Monoterpene						37.5±0.0
1132	Neo allo-ocimene	Monoterpene		26.3±2.0	3.1±0.6			52.4±0.8
1137	(E,Z)-Ethyl 2,4-hexadienoate	Ester					5.1±1.7	
1137	Camphor	Monoterpene				24.2±5.7		
1142	Isopinocarveol	Monoterpene		15.4±2.3				

1147	2-Nonenal	Aldehyde		9.4±3.2			4.0±0.3	4.2±4.2
1147	Nopinone	Ketone				1.9±0.9		6.1±6.1
1153	(E,Z)-2,6-Nonadienal	Aldehyde					10.3±7.0	
1153	Veratrol	Phenolic				4.8±1.0		
1153	Nonanol	Alcohol	12.9±2.4	15.9±2.3				43.3±6.2
1158	Isoborneol	Monoterpene						32.4±11.4
1158	Sabina ketone	Ketone				5.8±0.6		
1158	Homofuraneol	Furanone			0.8±0.9			
1163	γ-Terpineol, dihydro-	Monoterpene				4.0±2.2		8.0±8.0
1168	3-Ethylphenol	Phenolic						4.6±4.6
1168	(-)-Borneol	Monoterpene				4.0±0.1		27.3±4.0
1168	p-Cymen-8-ol	Monoterpene		8.3±1.1				
1168	α-Phellandren-8-ol	Monoterpene			3.4±1.0			
1174	(E)-Linalool oxide, trans	Monoterpene		19.3±10.1		8.1±1.5	180±30.6	9.1±9.1
1179	4-Terpinenol	Monoterpene	25.9±1.2	116.6±13.8	8.5±2.3	12.3±3.0		173.7±13.7
1184	Ethyl benzoate	Ester	283.5±31.6					
1184	Hexyl butanoate	Ester				112.5±64.9		54.5±3.5
1195	Myrtenol	Monoterpene		365.0±184.0		1061.2±102.5		
1197	α-Terpineol	Monoterpene	697.6±13.5	753.4±70.8	40.4±7.2	36.7±9.7	56.8±2.5	43.8±1.7
1200	Ethyl octanoate	Ester		221.2±23.0				
1203	Verbenone	Monoterpene			29.3±3.3	44.0±5.2		
1203	Carveol	Monoterpene					16.7±2.6	
1206	cis-Isocarveol	Monoterpene			5.4±0.3			9.9±3.0
1211	Decanal	Aldehyde	30.8±4.1	8.5±4.3	6.0±1.0	11.5±1.7	3.2±3.2	12.0±0.5
1214	Isobutyric acid	Acid				375.3±187.7		
1217	(-)-Myrtenol	Monoterpene		1990.4±61.8	434.4±172.6			384.8±64.1
1217	trans-Carveol	Monoterpene			8.4±0.6			
1217	(E,E)-2,4-Nonedienal	Aldehyde					17.8±2.0	
1220	Neodihydrocarveol	Monoterpene						20.8±3.1
1226	Cuminaldehyde	Aldehyde		496.1±255.1	25.3±4.5			
1226	Nerol	Monoterpene	63.5±10.7	39.3±4.3	14.9±0.8		13.8±1.4	3.0±3.0

1229	cis-Carveol	Monoterpene			3.8±0.5			
1229	Methyl nonanoate	Ester			3.7±0.8			
1229	Verbenone	Monoterpene						16.0±0.4
1231	Citronellol	Monoterpene	247.7±17.1					
1234	Methyl salicylate	Ester				5.0±2.7		
1237	Pulegone	Monoterpene					7.6±0.8	
1237	(-)-Myrtenal	Monoterpene			2.0±2.4			
1240	4-Phenyl-2-Butanol	Alcohol			11.6±0.1			
1240	cis-Citral; Neral	Monoterpene		159.3±33.9				
1240	Benzothiazole	Benzothiazole				6.7±0.9		
1243	(E)-4-tert-butylcyclohexanol	Alcohol	26.3±8.6					
1246	Piperitone	Monoterpene			4.4±0.9			
1254	Carvone	Monoterpene	26.0±3.5	6.0±3.1	3.3±0.7			10.9±0.9
1257	Isobornyl acetate	Ester		9.8±3.8		10.8±1.8		
1260	γ-octalactone	Lactone			12.0±1.0	8.8±1.1		55.1±27.0
1263	Phenylacetic acid	Acid			17.1±3.5			
1266	1-Decanol	Alcohol		28.7±4.7				
1269	Isopiperitenone	Monoterpene			3.4±0.2			
1269	5-Hydroxymethylfurfural	Furan		38.5±19.3				39.2±8.8
1271	Phellandral	Monoterpene		69.3±34.7				
1274	Geraniol	Monoterpene	15.5±1.2	38.2±19.9	9.7±0.9		11.9±0.1	
1280	Octanoic acid	Acid			3.4±0.1			
1280	Vitispirane I II	C13 Norisoprenoid						15.5±2.9
1283	Cumin alcohol	Alcohol			1.9±0.2			
1283	Cinnamaldehyde	Aldehyde		8.3±4.1				20.8±1.0
1289	δ-octalactone	Lactone			3.6±0.4			
1289	Bornyl acetate	Ester						22.9±2.1
1294	Perilly alcohol	Alcohol		4.9±2.4	10.5±4.7			4.3±4.3
1294	Undecanal	Aldehyde			10.2±1.2	6.9±1.5		
1297	2-Undecanone	Ketone		23.2±2.3	15.6±10.5	22.3±6.3		

1300	Butyrolactone	Lactone	14.7±3.4				
1300	Theaspirane I (A)	C13 Norisoprenoid		27.0±5.8			7.2±1.8
1313	Cinnamic alcohol	Alcohol		8.1±1.3		5.7±1.6	
1313	Theaspirane II (B)	C13 Norisoprenoid	30.6±1.6	10.9±1.6			11.8±0.4
1319	Myrtenyl acetate	Ester	5.8±3.4	5.4±3.4			
1325	Methyl decanoate	Ester			10.7±2.4		20.3±3.7
1338	d-Elemene	Sesquiterpene				8.6±2.2	
1338	Methyl anthranilate	Ester	13.7±4.1	9.3±2.3	7.5±0.6		
1350	α-Cubebene	Sesquiterpene	9.6±3.7				
1350	2-undecenal	Aldehyde		53.6±1.2			
1356	Citronellyl acetate	Ester		4.8±0.3			
1363	Eugenol	Monoterpene	15.4±2.4	138.0±3.1	11.1±1.7	34.5±0.1	
1369	α-Copaene	Sesquiterpene	14.7±5.3				
1369	Undecan-2-ol	Alcohol			7.1±1.6		
1381	Geranyl acetate	Ester				22.3±14.6	
1369	Heptadecane, 9-hexyl-	Hydrocarbon		6.1±1.4			
1375	Decanoic acid	Acid			12.1±2.8	10.1±2.7	
1375	1-Undecanol	Alcohol		1.0±0.6			
1388	1,2-Oxolinalool	Monoterpene		36.7±6.9			
1388	Damascenone	C13 Norisoprenoid	21.8±3.4			39.3±9.2	
1394	α-Ionol	C13 Norisoprenoid	20.6±6.2	43.5±16.5			
1400	Ethyl decanoate	Ester					13.0±1.7
1407	Dodecanal	Aldehyde	11.9±7.0				
1407	Homovanillyl alcohol	Alcohol		4.7±0.8			
1413	Vanillin	Phenolic	14.0±6.9	62.8±4.8	5.8±1.3	32.0±0.8	
1420	α-ionone	C13 Norisoprenoid	17.6±9.1	69.7±2.6			
1447	(E)-Geranyl acetone	Monoterpene		2.4±1.5		3.4±0.1	

[1]Mean values (n=3) ±SEM.

1433	Dihydro-beta-ionone	C13 Norisoprenoid	7.6±2.3	8.1±2.4	9.1±0.9		
1453	Dihydro-beta-ionol	C13 Norisoprenoid	5.9±0.6	6.8±1.4			
1460	Isogeraniol	Monoterpene		21.9±12.7			
1467	Trans-β-Caryophyllene	Sesquiterpene	9.7±1.4				
1493	β-ionone	C13 Norisoprenoid	9.2±0.9	20.3±3.2	2.6±0.2	56.9±0.0	56.3±56.3
1513	Raspberry ketone	Ketone	54.8±3.9	36.8±3.5			
1520	δ-Cadinene	Sesquiterpene	9.4±0.8				
1520	δ-decalactone	Lactone		8.2±0.2			
1607	γ-undecalactone	Lactone	9.7±0.5				
1615	3-Hydroxy-β-damascone	C13 Norisoprenoid	8.5±4.3				
1630	3-Oxo-α-ionol	C13 Norisoprenoid	4.2±2.1				
1637	Zingerone	Phenolic	5.1±1.2	16.0±3.9			
1724	δ-Dodecalactone	Lactone		7.1±2.2			
